# *Liberomycespistaciae* sp. nov., the causal agent of pistachio cankers and decline in Italy

**DOI:** 10.3897/mycokeys.40.28636

**Published:** 2018-09-18

**Authors:** Salvatore Vitale, Dalia Aiello, Vladimiro Guarnaccia, Laura Luongo, Massimo Galli, Pedro W. Crous, Giancarlo Polizzi, Alessandra Belisario, Hermann Voglmayr

**Affiliations:** 1 Consiglio per la Ricerca in Agricoltura e l’Analisi dell’Economia Agraria (CREA)- Centro di Ricerca Difesa e Certificazione (DC), Via C. G. Bertero 22, 00156 Roma, Italy Centro di Ricerca Difesa e Certificazione Roma Italy; 2 Dipartimento di Agricoltura, Alimentazione e Ambiente, sezione Patologia Vegetale, University of Catania, Via S. Sofia 100, 95123 Catania, Italy University of Catania Catania Italy; 3 Westerdijk Fungal Biodiversity Institute, Uppsalalaan 8, 3584 CT Utrecht, The Netherlands Westerdijk Fungal Biodiversity Institute Utrecht Netherlands; 4 Department of Plant Pathology, University of Stellenbosch, Matieland 7602, South Africa University of Stellenbosch Matieland South Africa; 5 Division of Systematic and Evolutionary Botany, Department of Botany and Biodiversity Research, University of Vienna, Rennweg 14, 1030 Wien, Austria University of Vienna Vienna Austria

**Keywords:** Delonicicolaceae, nut disease, pathogenicity, *
Pistacia vera
*, Xylariales, 1 new species

## Abstract

A new canker and decline disease of pistachio (*Pistaciavera*) is described from Sicily (Italy). Observations of the disease and sampling of the causal agent started in spring 2010, in the area where this crop is typically cultivated, Bronte and Adrano (Catania province) and later extended to the Agrigento and Caltanissetta provinces. Isolations from the margins of twig, branch and stem cankers of declining plants resulted in fungal colonies with the same morphology. Pathogenicity tests on 5-year-old potted plants of *Pistaciavera* grafted on *P.terebinthus* reproduced similar symptoms to those observed in nature and the pathogen was confirmed to be a coloniser of woody plant tissue. Comparison of our isolates with the type of the apparently similar *Asteromellapistaciarum* showed that our isolates are morphologically and ecologically different from *A.pistaciarum*, the latter being a typical member of Mycosphaerellaceae. *Asteromellapistaciarum* is lectotypified, described and illustrated and it is considered to represent a spermatial morph of *Septoriapistaciarum*. Multi-locus phylogenies based on two (ITS and LSU rDNA) and three (ITS, *rpb2* and *tub2*) genomic loci revealed isolates of the canker pathogen to represent a new species of *Liberomyces* within the Delonicicolaceae (Xylariales), which is here described as *Liberomycespistaciae***sp. nov.** (Delonicicolaceae, Xylariales). The presence of this fungus in asymptomatic plants with apparently healthy woody tissues indicates that it also has a latent growth phase. This study improves the understanding of pistachio decline, but further studies are needed for planning effective disease management strategies and ensuring that the pathogen is not introduced into new areas with apparently healthy, but infected plants.

## Introduction

Cases of pistachio tree decline with gummosis, leaf canopy thinning and fruit losses have been observed for several years in the area of Bronte (Catania province, Sicily, Italy), which is considered the most typical area where high-quality pistachios are produced in Italy (http://www.dibartolosrl.it/bronte-pistachios/). Although pistachio is characterised by good rusticity, it is subject to several fungal diseases known to afflict pistachio trees in the Mediterranean area. Of these, the most commonly reported are phylloptosis, leaf spots mainly caused by *Septoriapistaciae*, *S.pistaciarum* and *Pseudocercosporapistacina*, gum cankers by *Cytosporaterebenthi* and branch and twig cankers by *Botryosphaeriadothidea* (Chitzanidis 1995, Teviotdale et al. 2002, Vitale et al. 2007, Crous et al. 2013). The latter is widespread and already present as a latent pathogen in numerous plant communities in various parts of the world (Marsberg et al. 2017). Amongst soil-borne pathogens, *Verticilliumdahliae* and *Phytophthora* spp. are reported to be particularly damaging in California (Holtz 2008). Moreover, recently a new blight was reported on pistachio fruit caused by *Arthriniumxenocordella* in the Agrigento province, southern Italy (Aiello et al. 2018).

From spring 2010 onwards, surveys have been carried out in 15 pistachio orchards of Catania, Agrigento and Caltanissetta provinces, Sicily, where declining trees were present. Declining plants showed twig, branch and stem cankers associated with vascular necrosis and tree decline. Abundant gummosis often occurred in association with cankered lesions. The cankered area resulted in localised, sunken lesions with several central cracks. After removing the bark, discolouration and necrotic tissue were evident and lesions deepened into the woody tissue. A coelomycetous fungus with pycnidial conidiomata was consistently isolated from these lesions.

The aims of this study were thus to investigate the aetiology of the decline syndrome observed in Bronte and to provide morphological, taxonomic, phylogenetic and pathogenic evidence of the causal organism which proved to be an undescribed species of *Liberomyces*, which was initially misidentified as *Asteromellapistaciarum*.

## Materials and methods

### Field survey and isolation

**Figure 1.**
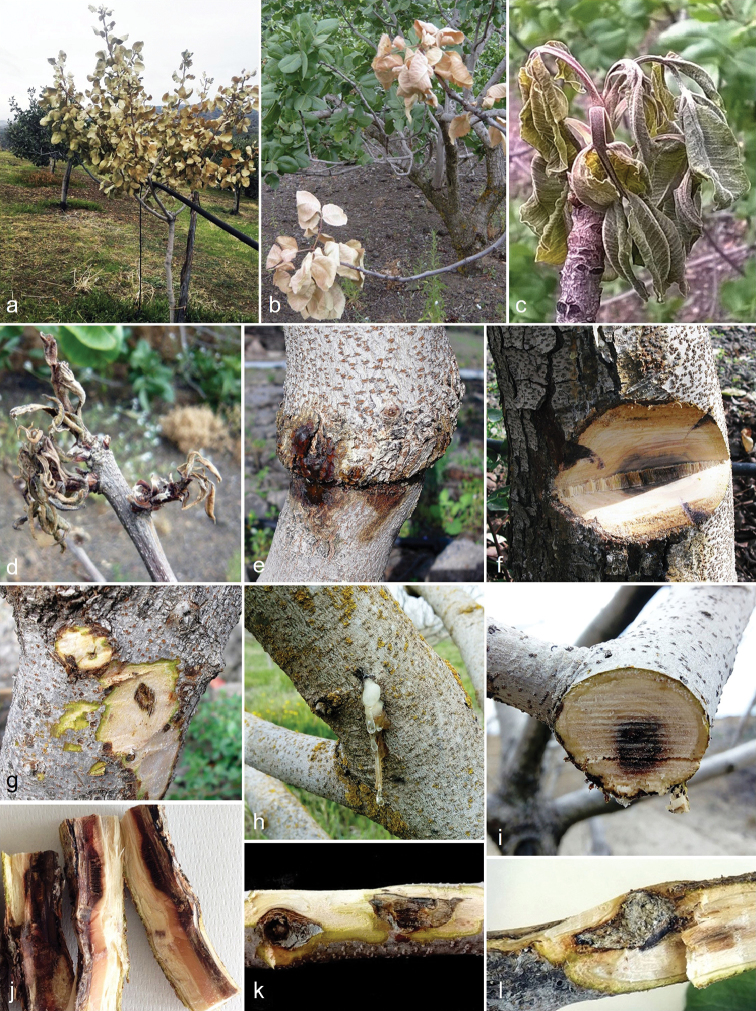
Symptoms caused by *Liberomycespistaciae* on *Pistaciavera in vivo*. **a** Plant killed by canker on trunk **b** Twigs dieback **c, d** Shoots wilted on infected twig **e** Gum and cracking of the trunk **f, g** Internal tissue of trunk cankers **h** Gum exudation on branch **i** Internal dark discolouration in cross section of branch **j** Necrotic tissue in longitudinal section of twig **k, l** External and internal cankers on twigs.

Surveys of 15 pistachio orchards were conducted from 2010 to 2017 in Bronte and Adrano (Catania province, eastern Sicily) and Agrigento and Caltanissetta provinces (western Sicily). Approximately 10 samples per orchard showing cankered twigs and branches from declining pistachio plants were randomly collected for analysis (Fig. 1). Sub-cortical and wood fragments (about 5 × 5 mm) were cut from the lesion margins between affected and healthy tissues. In addition, from one orchard in Bronte, twigs were also sampled from asymptomatic pistachio plants. Subsequently, tissue pieces were disinfected by soaking in 70% ethanol for 5 s, 4% sodium hypochlorite for 90 s, rinsed in sterile water for 60 s and dried on sterile filter paper in a laminar flow cabinet. The fragments were placed on to 1.5% (w/v) malt extract agar (MEA, Oxoid, Basingstoke, UK) and 2% potato dextrose agar (PDA, Oxoid), incubated at room temperature (25 ± 5 °C) and examined for fungal growth. Numerous slow-growing cultures were obtained and single-conidial isolations were performed with conidia collected from pycnidia produced on those cultures within one month of incubation at room temperature under natural light conditions. More than 80 single-spore isolates were obtained from symptomatic and asymptomatic tissue isolations. Amongst these, 71 isolates were characterised by molecular and phylogenetic analysis (Table 1) and the four isolates ISPaVe1958, ISPaVe2105, ISPaVe2106 and ISPaVe2148 were considered for morphological, taxonomic and pathogenic studies. For a summary of sampling information of these isolates, see Suppl. Material 1.

### Morphological characterisation

For morphological investigations, cultures were grown on MEA, PDA and 2% corn meal agar (CMA, Sigma-Aldrich) supplemented with 2% w/v dextrose (CMD). Moreover, pycnidial formation was assessed on artificially inoculated sterilised pistachio twigs incubated in a moist chamber. The isolates used in this study are maintained in the culture collections of the Dipartimento di Agricoltura, Alimentazione e Ambiente, University of Catania (PV) and of the CREA-DC (ex CREA-PAV), the ex-type isolate ISPaVe1958 of the new pistachio pathogen was deposited at the Westerdijk Fungal Biodiversity Institute (CBS), Utrecht, The Netherlands and the holotype specimen in the Fungarium of the Department of Botany and Biodiversity Research, University of Vienna (WU).

For investigations of temperature-growth relationships of the new pistachio pathogen, the holotype isolate ISPaVe1958 and the more recent isolate ISPaVe2148 were used. Agar plugs (5 mm diam.) were taken from the edge of actively growing cultures on MEA and transferred on to the centre of 9 cm diam. Petri dishes containing 1.5% MEA. Three replicate plates were incubated at 10, 15, 20, 25, 30 and 35 °C in the dark and measurements were taken after 21 d at right angles along two lines intersecting the centre of the inoculum and the mean growth rates plus and minus the standard deviation were calculated.

The holotype isolate ISPaVe1958 (CBS 128196) of the new pistachio pathogen and the type specimens of *Asteromellapistaciarum* deposited in the Natural History Museum of Vienna (W) were morphologically examined. For light microscopy, squash mounts and hand sections of pycnidia were made using a razor blade and observed in tap water or in 3% KOH. Methods of microscopy included stereomicroscopy using a Nikon SMZ 1500 equipped with a Nikon DS-U2 digital camera and light microscopy with Nomarski differential interference contrast (DIC) using the compound microscope Zeiss Axio Imager.A1 equipped with a Zeiss Axiocam 506 colour digital camera. Images were captured and measured with NIS-Elements D v. 3.0 or with the Zeiss ZEN Blue Edition software. For certain images of pycnidia, the stacking software Zerene Stacker v. 1.04 (Zerene Systems LLC, Richland, WA, USA) was used. Measurements are reported as maximum and minimum in parentheses and the range representing the mean plus and minus the standard deviation of a number of measurements given in parentheses.

### Pathogenicity

Pathogenicity tests with four fungal strains of the undescribed pistachio pathogen were performed to satisfy Koch’s postulates. Trials were carried out outdoors and in a growth chamber at 25 ± 1 °C. Potted 5-yr-old plants of *Pistaciavera* grafted on to *P.terebinthus* were used for artificial inoculations. Three plants for each isolate and six inoculation sites for each plant were considered.

Inoculations were made on stems and twigs after removing a 5 mm diam. bark disc with a cork borer, replacing it with a 5 mm plug from a 14-d-old PDA culture and covering it with sterile wet cotton, wrapped with parafilm (Pechney Plastic Packaging Inc., Chicago, USA) and aluminium foil to prevent contamination and desiccation. An equivalent number of plants and inoculation sites were inoculated with sterile PDA plugs as controls. The inoculated plants were observed every week. Symptom typology and the length of lesions were assessed after 12 months. To fulfil Koch’s postulates, re-isolation was conducted following the same procedure as described above for isolations. Tissue fragments were plated on MEA or PDA and morphological and molecular identifications by sequencing the ITS rDNA were performed.

### DNA extraction, PCR amplification and sequencing

The extraction of genomic DNA from pure cultures was performed as reported in previous studies (Voglmayr and Jaklitsch 2011, Jaklitsch et al. 2012, Guarnaccia and Crous 2017) by using the DNeasy Plant Mini Kit (QIAgen GmbH, Hilden, Germany) or the Wizard Genomic DNA Purification Kit (Promega Corporation, WI, USA). For the ex-type strain of the new species, the complete internal transcribed spacer region (ITS1-5.8S-ITS2) and a ca. 0.9 kb fragment of the large subunit nuclear ribosomal DNA (nLSU rDNA) were amplified and sequenced as a single fragment with primers V9G (de Hoog and Gerrits van den Ende 1998) and LR5 (Vilgalys and Hester 1990); the complete ITS region of the other strains was amplified with primers ITS5 and ITS4 (White et al. 1990); the RNA polymerase II subunit 2 (*rpb2*) gene was amplified with primers fRPB2-5F2 and fRPB2-7cR (Liu et al. 1999, Sung et al. 2007) or dRPB2-5f and dRPB2-7r (Voglmayr et al. 2016); and the beta-tubulin (*tub2*) gene with primer pairs T1 and T22 or Tub2Fd and Bt-2b (O’Donnell and Cigelnik 1997, Aveskamp et al. 2009). The PCR product was purified using an enzymatic PCR cleanup (Werle et al. 1994) as described in Voglmayr and Jaklitsch (2008). DNA was cycle-sequenced using the ABI PRISM Big Dye Terminator Cycle Sequencing Ready Reaction Kit v. 3.1 (Applied Biosystems, Warrington, UK) with the same primers as in PCR; in addition, primers ITS4, LR2R-A (Voglmayr et al. 2012) and LR3 (Vilgalys and Hester 1990) were used for the ITS-LSU fragment. Sequencing was performed on an automated DNA sequencer (3730xl Genetic Analyser, Applied Biosystems).

### Phylogenetic analyses

NCBI BLASTn searches of the ITS and LSU of the undescribed pistachio pathogen revealed members of Xylariales as closest matches. For phylogenetic analyses, two combined matrices were produced; GenBank accession numbers of the sequences used in the phylogenetic analyses are given in Table 1. A combined ITS-LSU matrix was generated to reveal the phylogenetic position of the undescribed pistachio pathogen within Xylariales. For this, representative GenBank sequences of Xylariales were selected from Jaklitsch et al. (2016) and supplemented with some additional GenBank sequences; six taxa of Sordariomycetes were added as outgroup. The second combined matrix contained three loci (ITS, *rpb2*, *tub2*) sequenced for 68 isolates of the undescribed pistachio pathogen; in addition, GenBank sequences of four accessions of Delonicicolaceae and of six additional members of Xylariales were added and two species of Diaporthales were used as outgroup (Guarnaccia and Crous 2017, Voglmayr et al. 2017).

All alignments were produced with the server version of MAFFT (www.ebi.ac.uk/Tools/mafft), checked and refined using BioEdit v. 7.0.9.0 (Hall 1999). After exclusion of ambiguously aligned regions and long gaps, the final ITS-LSU matrix contained 1340 nucleotide characters and the three loci matrix 1941 nucleotide characters (660 from ITS, 781 from *rpb2* and 500 from *tub2*). The alignment and phylogenetic trees were deposited in TreeBASE (http://purl.org/phylo/treebase/phylows/study/TB2:S23059).

Maximum Likelihood (ML) analyses were performed with RAxML (Stamatakis 2006) as implemented in raxmlGUI v. 1.3 (Silvestro and Michalak 2012), using the ML + rapid bootstrap setting and the GTRGAMMAI substitution model with 1000 bootstrap replicates.

Maximum Parsimony (MP) analyses were performed with PAUP v. 4.0a161 (Swofford 2002), using 1000 replicates of heuristic search with random addition of sequences and subsequent TBR branch swapping (MULTREES option in effect, steepest descent option not in effect, COLLAPSE command set to MINBRLEN). Molecular characters were unordered and given equal weight; gaps were treated as missing data. Bootstrap analyses with 1000 replicates were performed in the same way, with 5 rounds of replicates of heuristic search with random addition of sequences and subsequent TBR branch swapping during each bootstrap replicate, with each replicate limited to 1 million rearrangements in the analysis of the three-loci matrix.

## Results

### Field survey and isolation

Cankers and decline symptoms caused by the undescribed pistachio pathogen were detected in 10 orchards amongst the 15 investigated. The disease was primarily observed in the winter period and during late spring.

**Figure 2.**
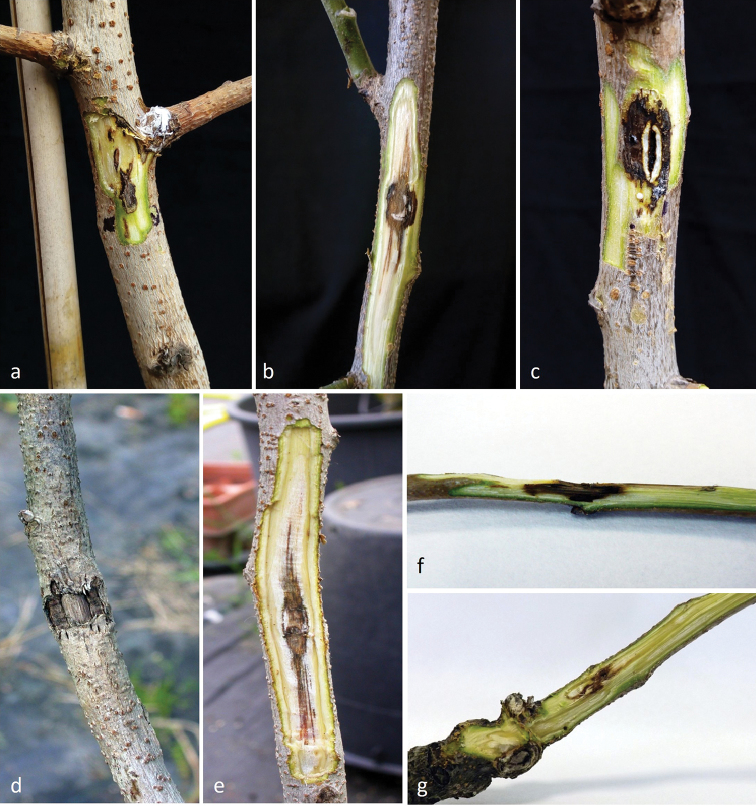
Symptoms reproduced from mycelial plug inoculation with *Liberomycespistaciae* on 5-year-old potted plants of *Pistaciavera*. Stem symptoms after **a, b** 3 wks **c** 6 months **d, e** 12 months **f, g** Cankers on twigs.

In the Bronte and Adrano areas (Catania province), symptoms were observed during the dormant season. Symptomatic plants showed gum exudation and often bark scaling on trunk and/or branches. When bark scaling occurred, it appeared as cracking and peeling of the bark. On trunks and large branches, cankers first appeared as visible dead circular areas that developed in the bark, which subsequently became dark and sunken. From that point onwards, infected areas expanded in all directions but much faster along the main axis of the stem, branch or twig. Under some environmental conditions, the host produced callus tissue around dead areas limiting the canker. Under the bark, cankers were characterised by discolouration and necrotic tissues and, in some cases, these extended to the vascular tissues and pith (Figs 1, 2).

During the active growing season, the symptomatic plants also showed canopy decline. Inflorescences and shoots, originating from infected branches or twigs, wilted and died. When the trunk was girdled by a canker, a collapse of the entire plant occurred (Fig. 1).

More than 80 single-spore isolates were obtained from symptomatic and a few also from asymptomatic pistachio plants. Amongst these, 71 isolates were characterised by molecular phylogenetic analyses and 68 deposited at the Westerdijk Fungal Biodiversity Institute, Utrecht, Netherlands (Table 1).

**Table 1.** Isolates and accession numbers used in the phylogenetic analyses.

Taxon	Strain^1,2,3^	ITS^3^	LSU^3^	*tub2* ^3^	*rpb2* ^3^
* Acrocordiella occulta *	CBS 140500^ET^	KT949893	KT949893		
* Alnecium auctum *	CBS 124263^ET^	KF570154	KF570154		
* Amphibambusa bambusicola *	MFLUCC 11-0617^HT^	KP744433	KP744474		
* Amphisphaeria umbrina *	HKUCC 994	AF009805	AF452029		
* Anthostoma decipiens *	CBS 133221	KC774565	KC774565		
* Arthrinium phragmites *	CBS 135458^HT^	KF144909	KF144956		
* Arthrinium saccharicola *	CBS 831.71	KF144922	KF144969		
* Barrmaelia macrospora *	CBS 142768^ET^	KC774566	KC774566		
* Bartalinia robillardoides *	CBS 122705^ET^	KJ710460	KJ710438	LT853252	LT853152
* Basiseptospora fallax *	CBS 129020^ET^	JF440983	JF440983		
* Beltrania rhombica *	CBS 141507	KX306749	KX306778		
* Beltraniella odinae *	NBRC 6774	00677401^4^	00677401^4^		
* Beltraniopsis neolitseae *	CBS 137974^HT^	KJ869126	KJ869183		
* Biscogniauxia nummularia *	MUCL 51395^ET^	JX658444	KT281894		
* Broomella vitalbae *	CBS 140412	KT949895	KT949895		
* Cainia graminis *	CBS 136.62	KR092793	AF431949		
* Calosphaeria pulchella *	CCTU 316	JX876610	JX876611		
* Camillea obularia *	ATCC 28093	KY610384	KY610429		
* Chaetosphaeria innumera *	MR 1175	AF178551	AF178551		
* Coniocessia maxima *	CBS 593.74^HT^	GU553332	GU553344		
* Coniocessia nodulisporioides *	CBS 281.77^IT^	GU553333	GU553352		
* Creosphaeria sassafras *	CBS 127876	KT949900	KT949900		
* Cryptovalsa rabenhorstii *	CBS 125574	KC774567	KC774567		
* Daldinia concentrica *	CBS 113277	AY616683	KT281895	KC977274	KY624243
* Delonicicola siamense *	MFLUCC 15-0670^HT^	MF167586	MF158345	–	MF158346
* Diaporthe eres *	CBS 109767	KC343075	AF408350		
* Diaporthe limonicola *	CBS 142549^HT^	MF418422		MF418582	**MH797629**
* Diatrype disciformis *	CBS 197.49	-	DQ470964		
* Discosia artocreas *	NBRC 8975	AB594773	AB593705		
* Eutypa lata *	CBS 208.87^NT^	DQ006927	DQ836903		
* Graphostroma platystoma *	CBS 270.87	JX658535	AY083827		
* Hymenopleella hippophaeicola *	CBS 140410^ET^	KT949901	KT949901		
* Hyponectria buxi *	UME 31430	-	AY083834		
* Hypoxylon fragiforme *	MUCL 51264^ET^	KC477229	KM186295		
* Idriella lunata *	MUCL 7551	KC775735	KC775710		
* Immersidiscosia eucalypti *	MAFF 242781	AB594793	AB593725		
* Juglanconis juglandina *	CBS 133343	KY427149		KY427234	KY427199
* Kretzschmaria deusta *	CBS 163.93	KC477237	KT281896		
* Leiosphaerella praeclara *	CBS 125586^ET^	JF440976	JF440976		
* Lepteutypa fuckelii *	CBS 140409^NT^	KT949902	KT949902		
* Liberomyces macrosporus *	CCF 4028^HT^	FR715522	FR715522	FR715498	FR715509
* Liberomyces pistaciae *	**CPC 31292 = CBS 144225**	**MH797562**		**MH797697**	**MH797630**
* Liberomyces pistaciae *	**CPC 31293**	**MH797563**		**MH797698**	**MH797631**
* Liberomyces pistaciae *	**CPC 31294**	**MH797564**		**MH797699**	**MH797632**
* Liberomyces pistaciae *	**CPC 31295**	**MH797565**		**MH797700**	**MH797633**
* Liberomyces pistaciae *	**CPC 31296**	**MH797566**		**MH797701**	**MH797634**
* Liberomyces pistaciae *	**CPC 31297**	**MH797567**		**MH797702**	**MH797635**
* Liberomyces pistaciae *	**CPC 31298**	**MH797568**		**MH797703**	**MH797636**
* Liberomyces pistaciae *	**CPC 31299**	**MH797569**		**MH797704**	**MH797637**
* Liberomyces pistaciae *	**CPC 31300**	**MH797570**		**MH797705**	**MH797638**
* Liberomyces pistaciae *	**CPC 31301**	**MH797571**		**MH797706**	**MH797639**
* Liberomyces pistaciae *	**CPC 31302**	**MH797572**		**MH797707**	**MH797640**
* Liberomyces pistaciae *	**CPC 31303**	**MH797573**		**MH797708**	**MH797641**
* Liberomyces pistaciae *	**CPC 31304**	**MH797574**		**MH797709**	**MH797642**
* Liberomyces pistaciae *	**CPC 31305**	**MH797575**		**MH797710**	**MH797643**
* Liberomyces pistaciae *	**CPC 31315**	**MH797576**		**MH797711**	**MH797644**
* Liberomyces pistaciae *	**CPC 31316**	**MH797577**		**MH797712**	**MH797645**
* Liberomyces pistaciae *	**CPC 31317**	**MH797578**		**MH797713**	**MH797646**
* Liberomyces pistaciae *	**CPC 31318**	**MH797579**		**MH797714**	**MH797647**
* Liberomyces pistaciae *	**CPC 31319**	**MH797580**		**MH797715**	**MH797648**
* Liberomyces pistaciae *	**CPC 31320**	**MH797581**		**MH797716**	**MH797649**
* Liberomyces pistaciae *	**CPC 31321**	**MH797582**		**MH797717**	**MH797650**
* Liberomyces pistaciae *	**CPC 31322**	**MH797583**		**MH797718**	**MH797651**
* Liberomyces pistaciae *	**CPC 31323**	**MH797584**		**MH797719**	**MH797652**
* Liberomyces pistaciae *	**CPC 31324**	**MH797585**		**MH797720**	**MH797653**
* Liberomyces pistaciae *	**CPC 31325**	**MH797586**		**MH797721**	**MH797654**
* Liberomyces pistaciae *	**CPC 31326**	**MH797587**		**MH797722**	**MH797655**
* Liberomyces pistaciae *	**CPC 31327**	**MH797588**		**MH797723**	**MH797656**
* Liberomyces pistaciae *	**CPC 31328**	**MH797589**		**MH797724**	**MH797657**
* Liberomyces pistaciae *	**CPC 31329**	**MH797590**		**MH797725**	**MH797658**
* Liberomyces pistaciae *	**CPC 31330**	**MH797591**		**MH797726**	**MH797659**
* Liberomyces pistaciae *	**CPC 31332**	**MH797592**		**MH797727**	**MH797660**
* Liberomyces pistaciae *	**CPC 31333**	**MH797593**		**MH797728**	**MH797661**
* Liberomyces pistaciae *	**CPC 33611**	**MH797594**		**MH797729**	**MH797662**
* Liberomyces pistaciae *	**CPC 33612**	**MH797595**		**MH797730**	**MH797663**
* Liberomyces pistaciae *	**CPC 33613**	**MH797596**		**MH797731**	**MH797664**
* Liberomyces pistaciae *	**CPC 33614**	**MH797597**		**MH797732**	**MH797665**
* Liberomyces pistaciae *	**CPC 33629**	**MH797598**		**MH797733**	**MH797666**
* Liberomyces pistaciae *	**CPC 33630**	**MH797599**		**MH797734**	**MH797667**
* Liberomyces pistaciae *	**CPC 33848**	**MH797600**		**MH797735**	**MH797668**
* Liberomyces pistaciae *	**CPC 33849**	**MH797601**		**MH797736**	**MH797669**
* Liberomyces pistaciae *	**CPC 33850**	**MH797602**		**MH797737**	**MH797670**
* Liberomyces pistaciae *	**CPC 33851**	**MH797603**		**MH797738**	**MH797671**
* Liberomyces pistaciae *	**CPC 33852**	**MH797604**		**MH797739**	**MH797672**
* Liberomyces pistaciae *	**CPC 33853**	**MH797605**		**MH797740**	**MH797673**
* Liberomyces pistaciae *	**CPC 33854**	**MH797606**		**MH797741**	**MH797674**
* Liberomyces pistaciae *	**CPC 33855**	**MH797607**		**MH797742**	**MH797675**
* Liberomyces pistaciae *	**CPC 33856**	**MH797608**		**MH797743**	**MH797676**
* Liberomyces pistaciae *	**CPC 33857**	**MH797609**		**MH797744**	**MH797677**
* Liberomyces pistaciae *	**CPC 33858**	**MH797610**		**MH797745**	**MH797678**
* Liberomyces pistaciae *	**CPC 33859**	**MH797611**		**MH797746**	**MH797679**
* Liberomyces pistaciae *	**CPC 33860**	**MH797612**		**MH797747**	**MH797680**
* Liberomyces pistaciae *	**CPC 33861**	**MH797613**		**MH797748**	**MH797681**
* Liberomyces pistaciae *	**CPC 33862**	**MH797614**		**MH797749**	**MH797682**
* Liberomyces pistaciae *	**CPC 33863**	**MH797615**		**MH797750**	**MH797683**
* Liberomyces pistaciae *	**CPC 33866**	**MH797616**		**MH797751**	**MH797684**
* Liberomyces pistaciae *	**CPC 33867**	**MH797617**		**MH797752**	**MH797685**
* Liberomyces pistaciae *	**CPC 33868**	**MH797618**		**MH797753**	**MH797686**
* Liberomyces pistaciae *	**CPC 33869**	**MH797619**		**MH797754**	**MH797687**
* Liberomyces pistaciae *	**CPC 33870**	**MH797620**		**MH797755**	**MH797688**
* Liberomyces pistaciae *	**CPC 33871**	**MH797621**		**MH797756**	**MH797689**
* Liberomyces pistaciae *	**CPC 33872**	**MH797622**		**MH797757**	**MH797690**
* Liberomyces pistaciae *	**CPC 33873**	**MH797623**		**MH797758**	**MH797691**
* Liberomyces pistaciae *	**CPC 33874**	**MH797624**		**MH797759**	**MH797692**
* Liberomyces pistaciae *	**CPC 34204**	**MH797625**		**MH797760**	**MH797693**
* Liberomyces pistaciae *	**CPC 34205**	**MH797626**		**MH797761**	**MH797694**
* Liberomyces pistaciae *	**CPC 34206**	**MH797627**		**MH797762**	**MH797695**
* Liberomyces pistaciae *	**CPC 34207**	**MH797628**		**MH797763**	**MH797696**
* Liberomyces pistaciae *	**ISPaVe1958^HT^ = CBS 128196**	**MH798901**	**MH798901**	**MH791335**	**MH791334**
* Liberomyces pistaciae *	**ISPaVe2105**	**FR681904**	–		
* Liberomyces pistaciae *	**ISPaVe2106**	**FR681905**	–		
* Liberomyces pistaciae *	**ISPaVe2148**	**MH798902**	–		
* Liberomyces saliciphilus *	H041	FR715510		FR715496	FR715507
* Liberomyces saliciphilus *	H077	FR715511		FR715497	FR715508
* Liberomyces saliciphilus *	CCF 4020^HT^	FR715515	FR715515		
* Lopadostoma turgidum *	CBS 133207^ET^	KC774618	KC774618		
* Melanconis stilbostoma *	CBS 121894	JQ926229	JQ926229		
* Melogramma campylosporum *	CBS 141086	JF440978	JF440978		
* Microdochium lycopodinum *	CBS 122885^HT^	JF440979	JF440979		
* Microdochium phragmitis *	CBS 285.71^ET^	AJ279449	EU926218	KP859076	KP859122
* Nectria cinnabarina *	CBS 125165^ET^	HM484548	HM484562		
* Neopestalotiopsis protearum *	CBS 114178^HT^	LT853103		LT853251	LT853151
* Pestalotiopsis knightiae *	CBS 114138^HT^	KM199310	KM116227		
* Phlogicylindrium eucalyptorum *	CBS 111689	KF251205	KF251708		
* Phlogicylindrium uniforme *	CBS 131312^HT^	JQ044426	JQ044445		
* Polyancora globosa *	CBS 118182^HT^	DQ396469	DQ396466		
* Poronia punctata *	CBS 656.78	KT281904	KY610496		
* Pseudapiospora corni *	CBS 140736^NT^	KT949907	KT949907		
* Pseudomassaria chondrospora *	CBS 125600	JF440981	JF440981		
* Pseudomassariella vexata *	CBS 129022^ET^	JF440977	JF440977		
* Requienella fraxini *	CBS 140475^HT^	KT949910	KT949910		
* Requienella seminuda *	CBS 140502^ET^	KT949912	KT949912		
* Robillarda sessilis *	CBS 114312^ET^	KR873256	KR873284		
* Sarcostroma restionis *	CBS 118154^HT^	DQ278922	DQ278924		
* Seimatosporium cupressi *	CBS 224.55^ET^	LT853083		LT853230	LT853131
* Seimatosporium rosae *	CBS 139823^ET^	KT198726	KT198727	LT853253	LT853153
* Seiridium marginatum *	CBS 140403^ET^	KT949914	KT949914		
* Seynesia erumpens *	SMH 1291	-	AF279410		
* Strickeria kochii *	CBS 140411^ET^	KT949918	KT949918		
* Truncatella angustata *	ICMP 7062	AF405306	AF382383		
* Vialaea insculpta *	DAOM 240257	JX139726	JX139726		
* Vialaea minutella *	BRIP 56959	KC181926	KC181924		
* Xylaria hypoxylon *	CBS 122620^ET^	KY610407	KY610495		
* Zetiasplozna acaciae *	CBS 137994^HT^	KJ869149	KJ869206		

^1^ Abbreviations: **ATCC**: American Type Culture Collection, Manassas, VA, USA**BRIP**: Queensland Plant Pathology Herbarium, Brisbane, Australia; **CBS**: Culture collection of the Westerdijk Fungal Biodiversity Institute, Utrecht, The Netherlands; **CCF**: Culture collection of the Dept. of Botany, Charles University, Prague, Czech Republic; **CCTU**: Culture Collection of Tabriz University, Iran; **CPC**: Culture collection of Pedro Crous, housed at CBS; H: Isolates from Pažoutová et al. (2012); **DAOM**: Canadian National Mycological Herbarium, Ottawa, Canada; **H**: Isolates from Pažoutová et al. (2012); **HKUCC**: The University of Hong Kong Culture Collection, Hong Kong, China; **ICMP**: International Collection of Microorganisms from Plants, Auckland, New Zealand; **ISPaVe**: Culture collection of the Consiglio per la Ricerca in Agricoltura e l’Analisi dell’Economia Agraria, Roma, Italy (CREA-DC); **MAFF**: MAFFGenbank, National Institute of Agrobiological Sciences, Ibaraki, Japan; **MFLUCC**: Mae Fah Luang University Culture Collection, Chiang Rai, Thailand; **MR**: Culture collection of Martina Réblová, Department of Taxonomy, Institute of Botany of the Czech Academy of Sciences, Průhonice, Czech Republic; **MUCL**: BCCM/MUCL Agro-food & Environmental Fungal Collection, Louvain-la-Neuve, Belgium; **SMH**: Culture collection of Sabine Huhndorf, Field Museum of Natural History, Chicago, USA; **UME**: Herbarium of the Department of Ecology and Environmental Science, Umeå University, Umeå, Sweden.

^2^**ET** Ex-epitype strain; **HT** Ex-holotype strain; **IT** Ex-isotype strain; **NT** Ex-neotype strain.

^3^ Isolates/sequences in bold were isolated/sequenced in the present study.

^4^ Sequence downloaded from NBRC (http://www.nbrc.nite.go.jp/).

### Pathogenicity

The initial symptom, observed 3 weeks after artificial fungal inoculation, was gum exudation produced around the point inoculated. After removing the bark, a dark discolouration and necrotic tissue were visible (Figs 2a, b). After 6 months, external cankers were observed in correspondence with the inoculated sites and small cracks were present in the sunken central area (Fig. 2c). After 12 months, symptoms were very obvious and similar to the cracked cankers observed in nature. Long and deep cracks were evident on the sunken area that defined the cankered lesion. After removing the bark, it was evident that the pathogen was able to colonise the wood and long discolourations were present (Figs 2d, e). After 12 months from inoculation, the length of lesions ranged from 12 to 45 mm. For ISPaVe1958 and ISPaVe2105, length lesions averaged 16.7 ± 3.0 and 31 ± 1.0 mm, respectively, while for ISPaVe2106 and ISPaVe2148, 18 ± 0.0 mm and 29.7 ± 2.0 mm. Controls measured 4.0 ±1.0 mm in average. Cultures, morphologically identical with the new pistachio pathogen, were re-isolated from these cankers, fulfilling Koch’s postulates. Moreover, ITS sequence comparison of these re-isolated cultures confirmed the species identity.

### Growth rates

**Figure 3.**
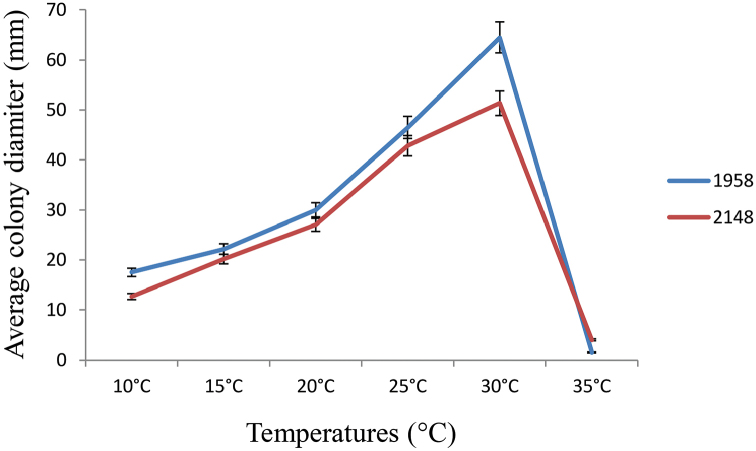
Temperature-growth relationships of the holotype isolate ISPaVe1958 compared to the more recent isolate ISPaVe2148 of *Liberomycespistaciae* on 1.5% MEA. Mean growth rates (mm) plus and minus the standard deviation, calculated on three replicates after 21 d of incubation, are shown.

The growth rate experiments revealed 30 °C as optimal temperature for both isolates with an evidently better growth of the holotype ISPaVe1958 at this temperature in comparison to ISPaVe2148 (Fig. 3).

### Phylogenetic analyses

**Figure 4.**
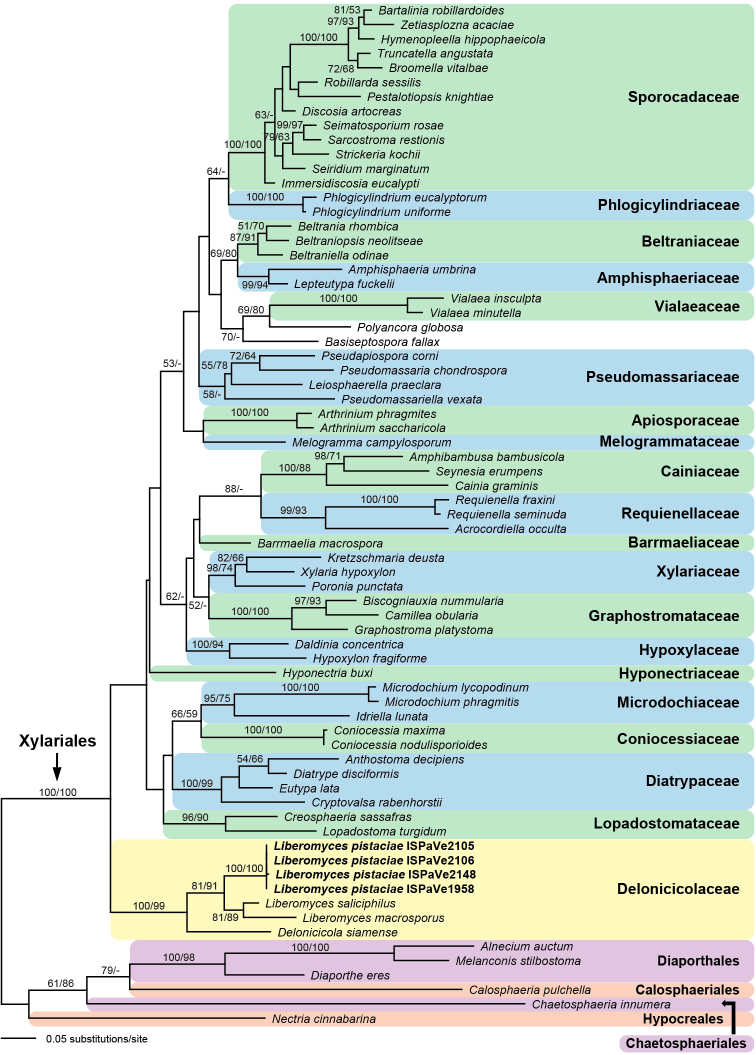
Phylogram of the best ML tree (-lnL = 19486.775) revealed by RAxML from an analysis of the combined ITS-LSU matrix of selected Xylariales, showing the phylogenetic position of *Liberomycespistaciae* (bold) within Delonicicolaceae. ML and MP bootstrap support above 50% are given above or below the branches.

Of the 1340 nucleotide characters of the ITS-LSU matrix, 519 are parsimony informative. The best ML tree (-lnL = 19486.775), revealed by RAxML, is shown as a phylogram in Fig. 4. Maximum parsimony analyses revealed 14 MP trees 4008 steps long (not shown). The backbone of the MP trees differs in several deeper unsupported nodes from the ML tree (not shown); notably in the MP tree, the *Liberomyces* clade was not the most basal node of Xylariales, although without support (not shown).

In the ML and MP analyses of the ITS-LSU matrix (Fig. 4), the Xylariales received maximum support, but backbone support within Xylariales was low to absent. The new species clustered within the *Liberomyces* clade, which was sister to *Delonicicolasiamense* (Delonicicolaceae, Xylariales). The Delonicicolaceae received high support (100% ML and 99% MP bootstrap support), but their closest relatives remained unclear due to lack of significant backbone support in all deeper nodes (Fig. 4). The sister-group relationship of *L.saliciphilus* and *L.macrosporus* received moderate support (81% ML and 89% MP bootstrap support).

**Figure 5.**
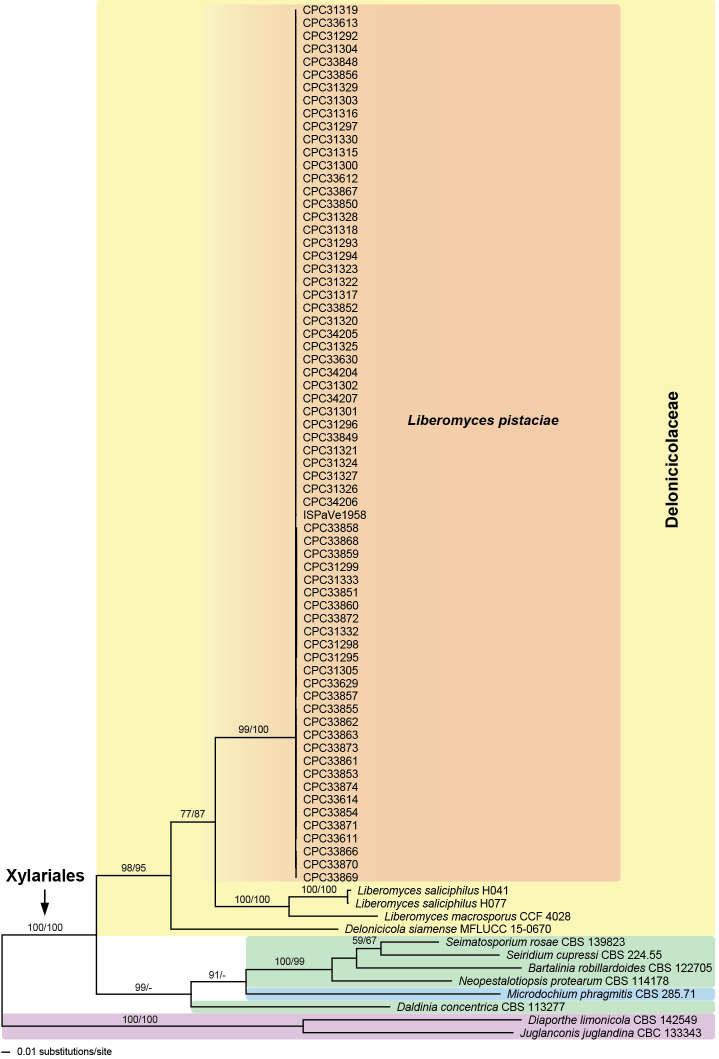
Phylogram of the best ML tree (-lnL = 12820.324) revealed by RAxML from an analysis of the combined ITS-*rpb2*-*tub2* matrix of selected Xylariales, showing the phylogenetic position of *Liberomycespistaciae* (bold) within Delonicicolaceae. The tree was rooted with two species of Diaporthales (*Diaporthelimonicola*, *Juglanconisjuglandina*). ML and MP bootstrap support above 50% are given above the branches.

Of the 1941 nucleotide characters of the ITS-*rpb2*-*tub2* matrix, 743 are parsimony informative (201 from ITS, 343 from *rpb2*, 199 from *tub2*). The best ML tree (-lnL = 12820.324), revealed by RAxML, is shown as a phylogram in Fig. 5. Maximum parsimony analyses revealed 6 MP trees 2669 steps long, with a tree backbone identical to that of the ML tree (not shown).

The analyses of the ITS-*rpb2*-*tub2* matrix (Fig. 5) revealed similar topologies to the analyses of the ITS-LSU matrix. The Xylariales and Delonicicolaceae received high support in ML and MP analyses. The new pistachio pathogen formed a genetically homogeneous clade with high to maximum support, confirming that all isolates sequenced belong to the same species. As in the ITS-LSU analyses, it was placed as sister to the highly supported *Liberomycessaliciphilus*-*L.macrosporus* clade with moderate support.

## Taxonomy

As a result of the morphological and molecular phylogenetic investigations, the undescribed pistachio pathogen is described as a new species, *Liberomycespistaciae*. In addition, for comparison, a morphological re-description and illustrations are also provided for the apparently similar, little known pistachio pathogen, *Asteromellapistaciarum*, based on type material and it is recognised as a synonym of *Septoriapistaciarum*.

### Liberomyces pistaciae

Taxon classificationFungiXylarialesDelonicicolaceae

Voglmayr, S. Vitale, D. Aiello, Guarnaccia, Luongo & Belisario sp. nov.

827682

Fig. 6

#### Diagnosis.

Species with distinctly smaller conidia (3.2–5.0 × 1.0–2.0 μm) than in *Liberomycessaliciphilus* Pažoutová, M. Kolařík & Kubátová and *L.macrosporus* Pažoutová, M. Kolařík & Kubátová.

#### Type.

ITALY. Sicily: Bronte (Catania province), on cankered twig of *Pistaciavera*, June 2010, A. Belisario (holotype: WU 39967; ex-type culture CBS 128196 = ISPaVe1958).

#### Etymology.

Named after its host genus, *Pistacia*.

#### Description.

*Conidiomata* pycnidial, superficial or immersed, single to densely aggregated, subglobose or cupular, uni- or irregularly plurilocular, first hyaline to pale brown, turning dark brown to blackish, without ostiole, irregularly rupturing at the apex and exuding a pale whitish conidial drop at maturity, (100–)170–260(–330) µm diam. (n=40). *Pycnidial wall* thin, of pale brown cells, (2.0–)3.5–6.3(–10.0) μm diam. (n=162) forming a *textura angularis*, outside darker, thicker-walled and more rounded, inside lined by a layer of angular hyaline cells giving rise to conidiophores. *Conidiophores* short, densely fasciculate, up to three times branched, hyaline, smooth, arising from the inner wall of the entire conidioma, 10–28 µm long. *Conidiogenous cells* holoblastic with sympodial proliferation, lageniform to cylindrical, (5.5–)6.5–8.5(–10.0) × 1.7–2.5(–2.7) µm (n=52), in dense intercalary or terminal whorls of 2–9. *Conidia* straight to allantoid, hyaline, smooth, 1-celled, (3.2–)3.8–4.5(–5.0) × (1.0–)1.2–1.5(–2.0) μm, l/w = (2.0–)2.7–3.5(–4.7) µm (n=182).

#### Culture characteristics.

**Figure 6.**
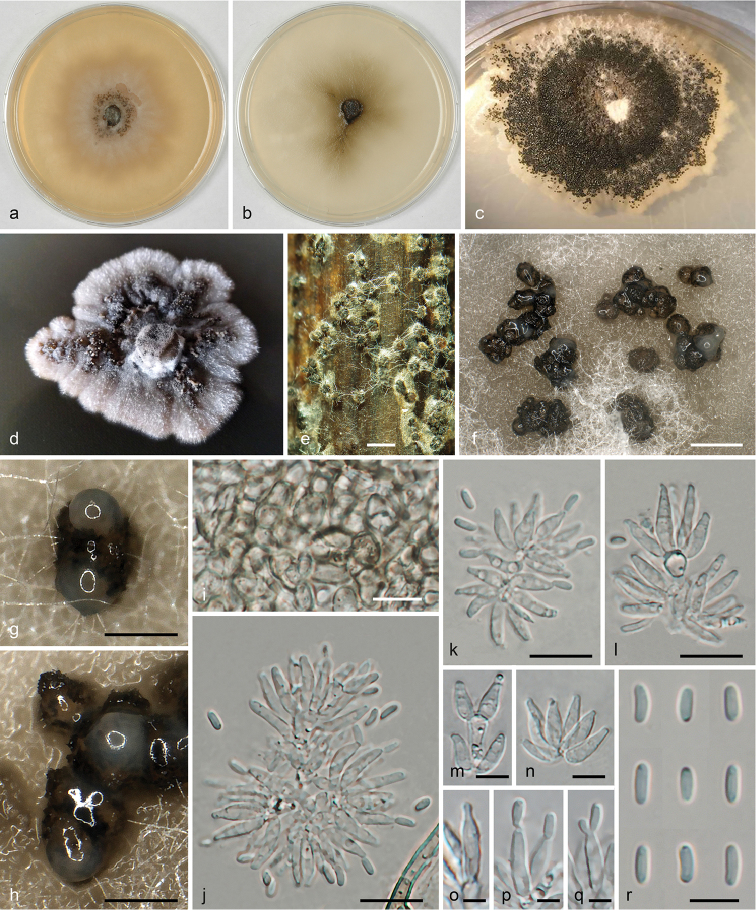
*Liberomycespistaciae*. **a–d** Cultures (**a**MEA, 6 weeks, 22 °C **b**CMD, 6 weeks, 22 °C **c**PDA, 3 weeks, 25 °C **d**PDA, 2 weeks, 25 °C) **e** Pycnidia produced on artificially inoculated sterilised pistachio twigs **f–h** Pycnidia in face view on MEA**i** Pycnidial wall in face view **j–n** Conidiophores and conidiogenous cells **o–q** Conidiogenous cells (**o** young **p, q** showing sympodial conidiation) **r** Conidia. All in water. Sources: **a–c, f–r** ex-holotype strain ISPaVe1958 = CBS 128196 **d, e** PV1= CPC 31292. Scale bars: 500 µm (**e, f**); 200 µm (**g, h**); 10 µm (**i–l**); 5 µm (**m–r**).

Colonies slow-growing (about 4 cm in diam. in 1 month on MEA, 4 cm in 2 weeks on CMD at 22 °C), initially white, turning pale to dark brown with age, with a whitish slightly lobed margin (Fig. 6a and b), surface mycelium sparse. Red to brown pigments diffusing in growth medium. Densely aggregated pycnidia formed after 7 d on the inoculum plug, successively also on the colony surface.

#### Notes.

Morphologically, *Liberomycespistaciae* is similar to the other two species of the genus, *L.macrosporus* and *L.saliciphilus*, but the latter have distinctly longer conidia (5–7.5 µm in *L.saliciphilus*, 8–13 µm in *L.macrosporus*).

### Septoria pistaciarum

Taxon classificationFungiXylarialesDelonicicolaceae

Caracc., Boll. Stud. Inform. R. Giard Colon Palermo 13: 10 [extr.] (1934).

#### Type

**of *Asteromellapistaciarum*.** TURKEY. Ankara, on leaves of *Pistaciavera*, 29 Oct. 1944, *H. Bremer* (lectotype of *Asteromellapistaciarum* designated here: W 1973-15537, MBT383208; isotype: W 1979-11134).

#### Description

**of the asteromella-like spermatial morph.***Infection* localised, producing distinct, brown, irregularly polyangular lesions of 0.5–1.5 mm diam., successively confluent, sharply delimited by leaf veins, visible on both sides of the leaf. *Pycnidia* (57–)69–101(–106) µm wide, (99–)107–134(–143) µm high (n=12), subepidermal, gregarious, solitary or in small groups, ellipsoid to pyriform, dark brown to black, with a central, circular, well-visible apical ostiole; peridium 8–19 µm wide, pseudoparenchymatous, of dark brown cells (3.0–)3.8–7.0(–10.3) µm diam. (n=50). Inner side lined with hyaline cells giving rise to phialides and short conidiophores. *Conidiophores* 1–3-celled, cells more or less square-shaped, bearing intercalary and terminal phialides. *Conidiogenous cells* enteroblastic, phialidic, hyaline, (3.7–)5.0–8.5(–10.5) × (2.5–)3.0–4.0(–4.7) µm (n=30), ampulliform to broadly lageniform, straight or curved. *Conidia* (3.4–)4.3–5.4(–6.6) × (0.9–)1.0–1.3(–1.5) μm, l/w = (2.8–)3.5–4.8(–6.1) (n=67), oblong, 1-celled, hyaline, with 1–2 subterminal guttules.

#### Notes.

The classification and description of the lectotype of *Asteromellapistaciarum* is here added as it is morphologically similar to *Liberomycespistaciae* and the latter had therefore initially been misidentified as the former (see e.g. Pažoutová et al. 2012, who included a sequence of *Liberomycespistaciae* as *Asteromellapistaciarum* in their phylogenies). In addition, *Asteromellapistaciarum* has not been addressed in previous taxonomic accounts. Two isotype specimens are located in the Natural History Museum of Vienna (W) from which W 1973-15537 is here selected as the lectotype based on preservation and abundance of the specimens. In the original description, Bremer and Petrak (1947) reported a close association of *Asteromellapistaciarum* with *Septoriapistaciarum* and an immature mycosphaerella-like sexual morph, which they consider to represent the same species. We agree with this treatment and consider *Asteromellapistaciarum* to be the spermatial morph of *Septoriapistaciarum*, the former therefore becoming a synonym of the latter based on priority.

**Figure 7.**
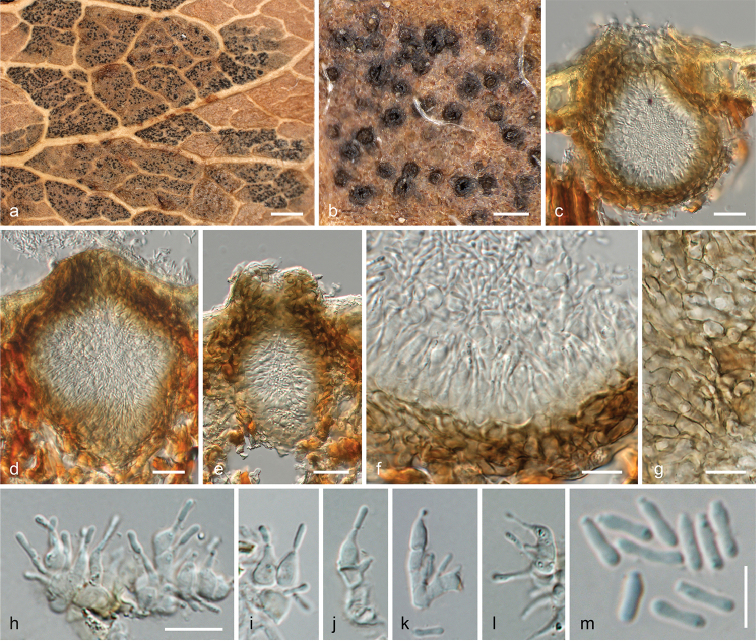
*Asteromellapistaciarum* W 1973-15537 (lectotype). **a, b** Pycnidia in leaf in face view **c–e** Pycnidia embedded in leaf in vertical section **f** Pycnidial wall with phialides and conidia in vertical section **g** Pycnidial wall in tangential section **h–l** Conidiophores and conidiogenous cells **m** Conidia. Scale bars: 10 mm (**a**); 100 µm (**b**); 20 µm (**c–e**); 10 µm (**f–l**); 5 µm (**m**).

## Discussion

This study represents the first work determining the causal agent of cankers and decline of pistachio trees in Sicily, the major production area of Italy. In the field, severe symptoms of canker were observed on branches, shoots and trunks. In some cases, decline and death of host plants also occurred. The fungus almost exclusively isolated from these symptoms was *Liberomycespistaciae* and the decline syndrome was strictly reproduced by artificial inoculation experiments. Seventy-one isolates recovered from different orchards over a 7-yr period were identified by molecular analysis. The molecular phylogenetic analyses (Figs 4, 5) clearly demonstrate that the genus *Liberomyces* is affiliated with the Xylariales, which confirms the results of previous analyses (Pažoutová et al. 2012, Perera et al. 2017). In both of our analyses, the genus *Liberomyces* is a sister group to *Delonicicolasiamense* with moderate to high support, for which Perera et al. (2017) established a new family and order, Delonicicolaceae and Delonicicolales. However, if the order Delonicicolales is accepted, the Xylariales are unsupported in Perera et al. (2017) as well as in our phylogenetic analyses of the ITS-LSU matrix (Fig. 4). In the order Xylariales, insufficient backbone resolution and support of phylogenies based on ITS-LSU rDNA has been commonly observed (e.g. Jaklitsch and Voglmayr 2012, Jaklitsch et al. 2016), which often significantly increases if protein-coding genes like *rpb2* and *tub2* are considered (e.g. Voglmayr et al. 2018, Wendt et al. 2018). However, for most lineages of Xylariales, only ITS-LSU rDNA data are currently available. Remarkably, also in the phylogenetic analyses of Perera et al. (2017), which were inferred from a combined SSU, ITS, LSU and *rpb2* matrix, internal support of Xylariales is absent if Delonicicolales are classified as a separate order. This fact may be due to lack of *rpb2* sequence data for many lineages within Xylariales. Therefore, we consider the establishment of a separate order Delonicicolales premature and presently we propose the classification of Delonicicolaceae within Xylariales in which this family also fits morphologically, given its conidiomatal morphology and conidiogenesis.

Due to the pycnidial conidiomata and conidia of similar sizes, the current pistachio pathogen, here described as *Liberomycespistaciae*, was initially identified as *Asteromellapistaciarum*, the true identity of which was unclear at that time. No sequence data are available for authentic material of the latter. However, a re-investigation of the type specimen of *A.pistaciarum* revealed substantial differences between both species, providing a clear distinction between the two organisms. While the type of *A.pistaciarum* has short reduced conidiophores with intercalary and terminal ampulliform phialides (Fig. 7h–l), *L.pistaciae* has densely fasciculate conidiophores with verticillately arranged holoblastic, lageniform to cylindrical conidiogenous cells with sympodial conidial proliferation (Fig. 6j–q). In addition, the type of *A.pistaciarum* has distinctly more elongate conidia with a l/w of (2.8–)3.5–4.8(–6.1), compared to (2.0–) 2.7–3.5(–4.7) in *L.pistaciae*. Moreover, the disease symptoms are markedly different. The type collection of *A.pistaciarum* represents a foliar pathogen causing clearly delimited polyangular leaf lesions with gregarious subepidermal pycnidia on both sides of the leaf (Fig. 7a), whereas *L.pistaciae* causes a canker disease of stems and branches. Although no recent collections, sequence data or cultures are available for *A.pistaciarum*, its close association with *Septoriapistaciarum* and an immature mycosphaerella-like sexual morph on the holotype specimen, which was already noted in the original description (Bremer and Petrak 1947), provides strong evidence that *A.pistaciarum* represents the spermatial morph of *Septoriapistaciarum* and it is therefore here considered to be a synonym of the latter.

There are many fungal genera which can act as plant pathogens, but may behave also as latent pathogens, while closely related species are symptomless endophytes (Carroll 1988). This is apparently also the case in the pathogen *Liberomycespistaciae*, which might have a latent phase within the host tissues since it was also isolated from asymptomatic pistachio plants. A latent phase represents a specific condition where the fungus can either develop symptoms or induce changes in the physiology of the host plant (Romero et al. 2001, Crous et al. 2015, 2016). Furthermore, Millar (1980) and Andrews et al. (1985) observed that certain latent pathogens become pathogenic when the host is stressed and this may be the case in *L.pistaciae* on pistachio trees in Bronte. In this regard, the ecology of its closest relatives, *L.macrosporus* and *L.saliciphilus*, is of interest, as they were isolated as bark and wood endophytes from several woody hosts (Pažoutová et al. 2012), indicating that the primary ecology of the genus *Liberomyces* may be endophytic, from which the pathogenic *L.pistaciae* may have evolved. However, detailed studies are necessary to evaluate the influence of stress on pathogenicity of *L.pistaciae*.

On the basis of the high disease incidence and the frequency of this species observed in several orchards in the last years, we believe that *L.pistaciae* represents a menace to pistachio production in Sicily. As no epidemiological data are yet available, it is not possible to suggest any control strategies to avoid *L.pistaciae* infections. Nevertheless, the use and distribution of infected propagation material taken from nurseries and mechanical injuries or pruning wounds could play an important role in promoting the infections. The recent increase in importance of this and other diseases of pistachio in Sicily has stimulated further research and studies are in progress to extend the survey to other areas and to obtain important information to formulate effective disease management strategies.

## Supplementary Material

XML Treatment for Liberomyces pistaciae

XML Treatment for Septoria pistaciarum

## Supplementary materials

Supplementary material 1 Information on *Liberomycespistaciae* isolates used in this study Data type: species data This is an open access article distributed under the terms of the Creative Commons Attribution License (CC BY 4.0), which permits unrestricted use, distribution, and reproduction in any medium, provided the original author and source are credited.Salvatore Vitale, Dalia Aiello, Vladimiro Guarnaccia, Laura Luongo, Massimo Galli, Pedro W. Crous, Giancarlo Polizzi, Alessandra Belisario, Hermann Voglmayr
