# Supplementary material for: Liberomycespistaciae sp. nov., the causal agent of pistachio cankers and decline in Italy
Source: MycoKeys. 2018 Sep 18;(40):29–51. doi: 10.3897/mycokeys.40.28636 (PMC6160797; doi:10.3897/mycokeys.40.28636)
Supplement: Supplementary material 1 — Information on Liberomycespistaciae isolates used in this study [file mycokeys-40-029-s001.docx]

Supplementary Table 1. Information on *Liberomyces pistaciae* isolates used in this study

| **Culture no. ^a^** | **Isolate code ^b^** | **Colletion year** | **Geographical location ^c^** |
| --- | --- | --- | --- |
| CBS 128196 | ISPaVe1958 | 2010 | Orchard 9, Bronte (CT) |
| n.a. | ISPaVe2105 | 2010 | Orchard 9, Bronte (CT) |
| n.a. | ISPaVe2106 | 2010 | Orchard 9, Bronte (CT) |
| n.a. | ISPaVe2148 | 2012 | Orchard 9, Bronte (CT) |
| CPC 31292 | PV1 | 2014 | Orchard 1, Bronte (CT) |
| CPC 31293 | PV2 | 2014 | Orchard 1, Bronte (CT) |
| CPC 31315 | PV3 | 2014 | Orchard 1, Bronte (CT) |
| CPC 31316 | PV4 | 2014 | Orchard 1, Bronte (CT) |
| CPC 31317 | PV5 | 2014 | Orchard 1, Bronte (CT) |
| CPC 31318 | PV6 | 2014 | Orchard 1, Bronte (CT) |
| CPC 31319 | PV7 | 2014 | Orchard 1, Bronte (CT) |
| CPC 31320 | PV8 | 2014 | Orchard 1, Bronte (CT) |
| CPC 31321 | PV9 | 2014 | Orchard 1, Bronte (CT) |
| CPC 31322 | PV10 | 2014 | Orchard 1, Bronte (CT) |
| CPC 31323 | PV11 | 2014 | Orchard 1, Bronte (CT) |
| CPC 31324 | PV12 | 2014 | Orchard 1, Bronte (CT) |
| CPC 31325 | PV13 | 2014 | Orchard 1, Bronte (CT) |
| CPC 31326 | PV14 | 2014 | Orchard 1, Bronte (CT) |
| CPC 31327 | PV15 | 2014 | Orchard 1, Bronte (CT) |
| CPC 31328 | PV16 | 2015 | Orchard 1, Bronte (CT) |
| CPC 31329 | PV17 | 2015 | Orchard 1, Bronte (CT) |
| CPC 31330 | PV18 | 2015 | Orchard 1, Bronte (CT) |
| CPC 31332 | PV20 | 2015 | Orchard 1, Bronte (CT) |
| CPC 31333 | PV21 | 2015 | Orchard 1, Bronte (CT) |
| CPC 33629 | PV22 | 2015 | Orchard 1, Bronte (CT) |
| CPC 33630 | PV24 | 2015 | Orchard 1, Bronte (CT) |
| CPC 33866 | PV25 | 2015 | Orchard 1, Bronte (CT) |
| CPC 33848 | PV26 | 2015 | Orchard 1, Bronte (CT) |
| CPC 33867 | PV27 | 2015 | Orchard 1, Bronte (CT) |
| CPC 33849 | PV28 | 2015 | Orchard 1, Bronte (CT) |
| CPC 33850 | PV29 | 2015 | Orchard 1, Bronte (CT) |
| CPC 31294 | PV30 | 2015 | Orchard 2, Bronte (CT) |
| CPC 31295 | PV31 | 2015 | Orchard 2, Bronte (CT) |
| CPC 33851 | PV32 | 2015 | Orchard 2, Bronte (CT) |
| CPC 33852 | PV33 | 2015 | Orchard 2, Bronte (CT) |
| CPC 31296 | PV34 | 2015 | Orchard 3, Bronte (CT) |
| CPC 31297 | PV35 | 2015 | Orchard 3, Bronte (CT) |
| CPC 33868 | PV36 | 2015 | Orchard 2, Bronte (CT) |
| CPC 33869 | PV37 | 2015 | Orchard 2, Bronte (CT) |
| CPC 33870 | PV38 | 2015 | Orchard 2, Bronte (CT) |
| CPC 33853 | PV39 | 2015 | Orchard 3, Bronte (CT) |
| CPC 33871 | PV40 | 2015 | Orchard 2, Bronte (CT) |
| CPC 33854 | PV41 | 2015 | Orchard 3, Bronte (CT) |
| CPC 33855 | PV42 | 2015 | Orchard 3, Bronte (CT) |
| CPC 33856 | PV44 | 2015 | Orchard 3, Bronte (CT) |
| CPC 33857 | PV45 | 2015 | Orchard 3, Bronte (CT) |
| CPC 33858 | PV46 | 2015 | Orchard 3, Bronte (CT) |
| CPC 33859 | PV47 | 2015 | Orchard 3, Bronte (CT) |
| CPC 33860 | PV48 | 2015 | Orchard 3, Bronte (CT) |
| CPC 33872 | PV49 | 2015 | Orchard 3, Bronte (CT) |
| CPC 31298 | PV50 | 2015 | Orchard 3, Bronte (CT) |
| CPC 31299 | PV51 | 2015 | Orchard 3, Bronte (CT) |
| CPC 31300 | PV53 | 2016 | Orchard 4, Adrano (CT) |
| CPC 31301 | PV54 | 2016 | Orchard 4, Adrano (CT) |
| CPC 31302 | PV55 | 2016 | Orchard 4, Adrano (CT) |
| CPC 31303 | PV56 | 2016 | Orchard 4, Adrano (CT) |
| CPC 31304 | PV57 | 2016 | Orchard 5, Bronte (CT) |
| CPC 31305 | PV58 | 2016 | Orchard 5, Bronte (CT) |
| CPC 33611 | PV60 | 2017 | Orchard 6, Bronte (CT) |
| CPC 33612 | PV61 | 2017 | Orchard 6, Bronte (CT) |
| CPC 33613 | PV62 | 2017 | Orchard 6, Bronte (CT) |
| CPC 33614 | PV63 | 2017 | Orchard 6, Bronte (CT) |
| CPC 33873 | PV64 | 2017 | Orchard 6, Bronte (CT) |
| CPC 33861 | PV65 | 2017 | Orchard 6, Bronte (CT) |
| CPC 33862 | PV66 | 2017 | Orchard 6, Bronte (CT) |
| CPC 33874 | PV67 | 2017 | Orchard 6, Bronte (CT) |
| CPC 33863 | PV69 | 2017 | Orchard 6, Bronte (CT) |
| CPC 34204 | PV76 | 2017 | Orchard 8, Bronte (CT) |
| CPC 34205 | PV77 | 2017 | Orchard 8, Bronte (CT) |
| CPC 34206 | PV78 | 2017 | Orchard 8, Bronte (CT) |
| CPC 34207 | PV79 | 2017 | Orchard 8, Bronte (CT) |

^a^ *CBS* Westerdijk Fungal Biodiversity Institute, Utrecht, The Netherlands; *CPC* Culture collection of P.W. Crous, housed at Westerdijk Fungal Biodiversity Institute, Utrecht, Netherlands; *n.a.* not applicable.

^b^ *ISPaVe* Culture collection housed at the Consiglio per la Ricerca in Agricoltura e l’Analisi dell’Economia Agraria (CREA-DC), Roma, Italy; *PV* Culture collection housed at the Dipartimento di Agricoltura, Alimentazione e Ambiente, University of Catania, Italy.

^c^ CT Catania province (Sicily, Italy).
